# Consumption of Butylated Starch Alleviates the Chronic Restraint Stress-Induced Neurobehavioral and Gut Barrier Deficits Through Reshaping the Gut Microbiota

**DOI:** 10.3389/fimmu.2021.755481

**Published:** 2021-09-17

**Authors:** Peijun Tian, Huiyue Zhu, Xin Qian, Ying Chen, Zheng Wang, Jianxin Zhao, Hao Zhang, Gang Wang, Wei Chen

**Affiliations:** ^1^State Key Laboratory of Food Science and Technology, Jiangnan University, Wuxi, China; ^2^School of Food Science and Technology, Jiangnan University, Wuxi, China; ^3^National Engineering Research Center for Functional Food, Jiangnan University, Wuxi, China; ^4^(Yangzhou) Institute of Food Biotechnology, Jiangnan University, Yangzhou, China; ^5^Wuxi Translational Medicine Research Center, Jiangsu Translational Medicine Research Institute, Wuxi, China

**Keywords:** butyrate, starch, depression, gut barrier, inflammation

## Abstract

The beneficial effect of short-chain fatty acids (SCFAs) on host health has been well recognized based on the booming knowledge from gut microbiome research. The role of SCFA in influencing psychological function is highlighted in recent years but has not been fully elucidated. In this study, the SCFA-acylated starches were used to accomplish a sizeable intestine-targeted release of the SCFAs, and the neurobehavioral, immunological, and microbial effects were further investigated. Acetylated-, butylated-, and isobutylated-starch could attenuate the depression-like behaviors and excessive corticosterone production in chronically stressed mice. Butylated- starch significantly reduced the colonic permeability *via* increasing the tight junction proteins (including ZO-1, Claudin, and Occludin) gene expression and reduced the level of the inflammatory cytokines (including IL-1β and IL-6). The butylated starch’s neurological and immunological benefits may be derived from the gut microbiome modifications, including normalizing the abundance of certain beneficial microbes (*Odoribacter* and *Oscillibacter*) and metabolomic pathways (*Tryptophan synthesis* and *Inositol degradation*). The present findings further validate the brain-beneficial effect of butyrate and offer novel guidance for developing novel food or dietary supplements for improving mental health.

## Introduction

Short-chain fatty acids (SCFAs) are the primary products of gut microbial fermentation from the undigested dietary fibers (1). Acetate, propionate, and butyrate (or isobutyrate) are the three major SCFAs (2). The regular communication between SCFAs and hosts participates in multiple physiological processes, including nutrients metabolism and immune system function (3). These SCFAs have been demonstrated to affect the host through multiple mechanisms: (a) SCFAs are energy sources for gastrointestinal mucosal cells. In particular, butyrate is the primary energy source for colonocytes (4). (b) SCFAs are the ligands of G Protein-Coupled Receptors (GPCR). The activation of GPR43 and GPR41 *via* SCFAs promotes the secretion of various hormones involving blood glucose maintenance, such as glucagon-like peptide-1 (GLP-1) and peptide tyrosine (PYY) (5, 6). (c) SCFAs could act as the histone deacetylases (HDACs) and therefore normalize the gene expression involving cell proliferation and anti-inflammatory process (6).

In recent years, growing evidence suggested that SCFAs are also a class of signaling molecules regulating brain function through the gut-brain axis (7). For example, feeding the obese mice with butyrate could activate the vagus nerve, which suppresses the appetite-related hormones’ release and then reduces the weight of mice (8). Besides, SCFAs secreted into the circulation could reach the brain and improve the blood-brain barrier’s permeability by up-regulating the tight junction protein expression (9). Direct intake of SCFAs also showed noticeable neuromodulation effects. A prior oral supplementation of the SCFA mixture (acetate, propionate, and butyrate) could prevent the mice from the neurobehavioral deficits induced by the repeated psychosocial stress, including depression and anxiety-like behaviors and impaired gut barrier function (10). In addition, our previous studies also proved that the SCFAs facilitate the biosynthesis of gut serotonin and the 5-hydroxytryptophan’s circulation, and this mechanism may account for the antidepressant-like effect of some psychobiotic strains such as the *Bifidobacterium longum* CCFM687 and *Bifidobacterium breve* CCFM1025 (11–13).

Previous research from our group indicated that intaking of SCFA-acylated starch, which results in increased SCFAs levels in the large intestine, alleviates the constipation symptoms in mice (14). All the evidence indicates the SCFAs possess great psychotropic potential. Inspired by the reported findings, here we use the SCFA-acylated starches as the dietary supplement to investigate the role of each SCFA in managing stress-induced neurobehavioral, immunological, and gut microbial abnormalities.

## Material and Methods

### Animal Experiment

**Figure 1:**
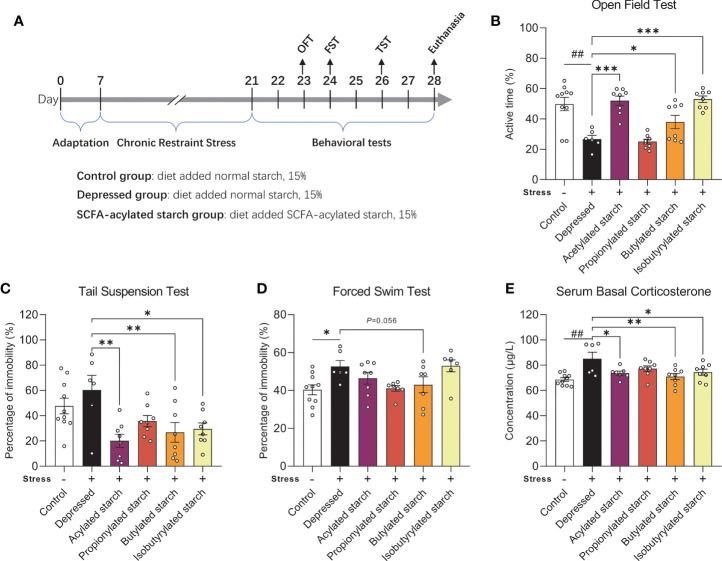
The effect of the SCFA-acylated starches on the neurobehavioral phenotype. **(A)** The animal experiment schedule. **(B)** Open field test. **(C)** Tail suspension test. **(D)** Forced swim test. **(E)** Serum corticosterone levels. Except for the control, all groups are given two-week chronic restraint stress. Data are mean with SEM (n=6-10 per group for each test). ^##^ *P*<0.01 in the unpaired t-tests. *P<0.05, **P<0.01, ***P<0.001 in the one-way ANOVA followed by Dunnett’s multiple comparisons test against the *Depressed* group.

The animal procedures were approved by the Ethics Committee of Experimental Animals at Jiangnan University [JN.No20190930c0501205(256)], following Directive 2010/63/EU guidelines. Animals were allowed to adapt to the environment (21°C~23°C, 50%~60% of humidity, 12:12h light-dark cycle) for seven days before the experiment. The group and experimental schedule are shown in **Figure 1A** (N=8-10 per group).

### Chronic Restraint Stress

The chronic restraint stress (CRS) paradigm was used to establish the depression-like phenotype (15). Mice were exposed to the restraint stress three h per day (from 09:00 to 15:00 every day, 1hour/each time, three times in total) for 14 consecutive days. Each mouse was put into a 50 mL plastic tube with a breathing hole and no food and water access. Mice in the control group fasted simultaneously during the CRS procedure. After the stress, all the mice were placed back in their home cages.

### Behavioral Tests

The protocols of open field test (OFT), tail suspension test (TST), and forced swim test (FST) were described in detail in our previously published papers (13). For the OFT, mice were assessed for their locomotor activity in a square arena (50 cm×50 cm) enclosed by continuous opaque walls made of plexiglass. Animals were monitored for 10 minutes, and the time spent in the center zone and active time during the whole period were measured. For the TST, animals were hanged using adhesive tape to fix the tails to a suspension bar (30 cm high from the floor). Locomotor activity is monitored for six minutes, and the duration of immobility was measured. For the FST, mice were allowed to swim in the water with a temperature of 23-25°C, and the immobility time was recorded during the ten-minute swimming. All mice are trained to swim for 15 minutes the day before the test. Before all the behavioral tests, animals were habituated to the room for one hour. All tests were performed under dim light (60 lux) and monitored by the video tracking system (Ethovision version 13, Noldus, Netherland).

### SCFA-Acylated Starches Preparation

SCFA-acylated starches were prepared as previously described (16, 17). Briefly, 40% (w/v) high amylose corn starch solution was continuously stirred in the water bath with a constant temperature of 40°. The pH was maintained at 8.00 by the addition of dilute NaOH (0.5 mol/L). Meanwhile, the acetic anhydride, propionic anhydride, butyric anhydride, and isobutyric anhydride were dripped into the starch solution, respectively, to reach a final amount of 20% (w/v). The solution was continued to be stirred for 2 hours, then adjusted to a pH of 5.70 using HCl (0.5 mol/L). The precipitation was collected by centrifuge (6000×g, 5min) and washed with water until the lotion was neutral. After a suction filtration at vacuum (-20 kPa, 10min), the precipitation was freeze-dried to get the SCFA-acylated starches. The starch solution without adding anhydride but processed as the same procedure above was used as control. The methods of measuring the degree of substitution of the SCFA-acylated starches and the results were described in **Supplemental 1.1**.

### SCFAs Determination

SCFAs levels were measured as previously described with some modifications (12, 18). Fecal samples from the distal ileum and cecum are weighed (about 50 mg), homogenized, and lyophilized. The samples were soaked in 500 μl of saturated NaCl solution for 30 min. The mixture was homogenized, and 40 μL 10% (w/v) sulfuric acid was added. 1 mL ether was added to the mixture and vortexed following centrifugation (18,000g, 15 min, 4°C). 500 μL supernatant was filtered (0.22 μm) before further analysis. SCFA concentrations were determined in the TSQ 9000 GC-MS system (Thermo Scientific) equipped with an Rtx-WAX capillary column, using helium as carrier gas (flow rate of 0.89 mL/min). A gradient profile of the oven temperature was used, starting at 100°C and increased to 140°C (7.5°C/min), then increased to 200°C (6.0°C/min) and maintained for 3 min. The ion source and interface temperature in the mass spectrometer were 220°C and 250°C, respectively.

### Hormones and Cytokines Determination

Serum corticosterone and inflammatory cytokines (TNF-α, IL-β, and IL-6) were measured using enzyme-linked immunosorbent assay (ELISA) kits according to the manufacturer’s protocol (SenBeiJia Biological Technology Co., Ltd., Nanjing, China).

### Gene Transcription of the Tight Junction Proteins

Total RNA of the colon tissues was extracted using Trizol reagent (Invitrogen, USA). Complementary DNA was prepared using the HiFiScript gDNA Removal cDNA Synthesis Kit (ComWin Biotech Co., Ltd., China). Transcription levels of the zonula occludens-1 (ZO-1), Claudin, and Occludin were determined by quantitative PCR. The gene expression was normalized to *Gapdh* based on the cycle threshold (Ct) values and the 2^−ΔΔCt^ method. All samples are measured in triplicate. Primer sets information are shown in **Supplemental Table S1**.

### 16S rRNA-Amplicon Sequencing of the Fecal Microbiome

16S rRNA gene amplicon sequencing of the fecal microbiome was performed using universal primers (341F/806R, for V3-V4 region) as previously described. Raw data were processed using the QIIME2 software package, and the specialized bioinformatic analysis was performed using the online software of MicrobiomeAnalyst (https://www.microbiomeanalyst.ca), including alpha diversity, beta diversity, and network analysis (19, 20). Gut metabolic modules (GMMs) analysis was performed using the R version of the Gomixer tool as previously described (21, 22). Detailed bioinformatical methods are described in **Supplemental 1.2**.

### Statistical Analysis

Data are presented as means with SEM. All data were checked for normality by the Shapiro-Wilk test. Unpaired Student’s t-test was performed between the *Control* and *Depressed* groups. One-way ANOVA followed by Dunnett’s multiple comparisons test against the *Depressed* group was performed to compare the effects of SCFA-acylated starches in the CRS-treated animals. A criterion for significance was set to *P*<0.05 in all comparisons. The *P*-value of multiple comparisons was adjusted by family-wise significance and confidence levels of 0.05 (95% confidence interval).

## Results

### Consumption of SCFA-Acylated Starches Changed the Depressive Symptoms of Stressed Mice

A two-week CRS induced significantly depressive behaviors, such as the significantly reduced active time in the OFT (*P*=0.002; **Figure 1B**) and increased immobility time in the FST (*P*=0.011; **Figure 1D**). The immobility time in the TST was increased but without any statistical difference (*P*=0.308; **Figure 1C**). Acetylated, butylated, and isobutylated starch reversed the animal’s behavioral abnormality in the OFT (**Figure 1B**; *P*<0.001, *P*=0.044, *P*<0.001, respectively) and TST (*P*=0.011, *P*=0.007, *P*=0.013, respectively; **Figure 1C**). Butylated starch also reduced the animal’s immobility time in the FST, with a trend statistical difference (*P*=0.056, **Figure 1D**). In addition, the stressed mice also showed a significantly reduced time spent in the center zone of the open field, but no SCFA-acylated starch reversed this abnormality (**Figure S1**). The hyperactivity of the hypothalamus-pituitary-adrenal axis was also observed in the stressed mice, as reflected by the significantly increased serum corticosterone level (*P*=0.002; **Figure 1E**). Acetylated, butylated, and isobutylated starch normalized the serum corticosterone level (**Figure 1E**; *P*=0.031, *P*=0.005, *P*=0.040, respectively).

### SCFA-Acylated Starches Increased the SCFA Levels in the Large Intestine

**Figure 2:**
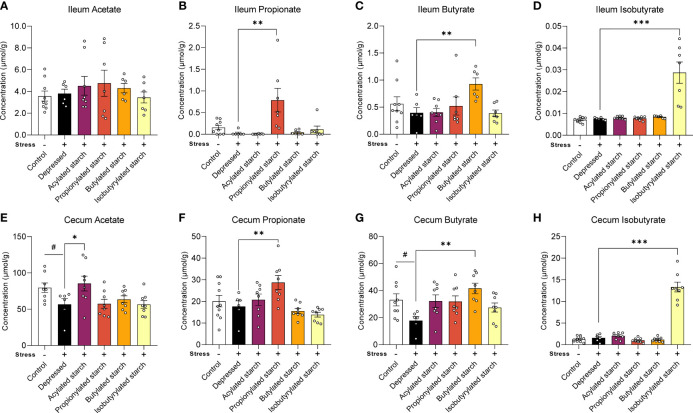
The SCFAs levels in the ileum and cecum. **(A–D)** Acetate, propitiate, butyrate, and isobutyrate levels in the ileum. **(E–H)** Acetate, propitiate, butyrate, and isobutyrate levels in the cecum. Except for the control, all groups are given two-week chronic restraint stress. Data are mean with SEM (n=6-10 per group for each test). ^#^ *P*<0.05 in the unpaired t-tests. *P<0.05, **P<0.01, ***P<0.001 in the unpaired t-tests.

In the distal ileum, the propionate (*P*=0.001), butyrate (*P*=0.009), and isobutyrate (*P*<0.001) levels were significantly increased when compared with the depressed mice, while the acetate level was not changed by the acetylated starch (**Figures 2A–D**). In the cecum, the acetate (*P*=0.044) and butyrate (*P*=0.024) levels were significantly decreased in the depressed mice, and the abnormalities were reversed by the acetylated (*P*=0.035) and butylated (*P*=0.001) starch treatment (**Figures 2E, G**). The cecum propionate (*P*=0.009) and isobutyrate (*P*<0.001) levels were also significantly increased when compared to the depressed mice (**Figures 2F, H**). All above data indicated that the SCFA-acylated starches resulted in a release of SCFAs in the gut, especially in the large intestine.

### Butylated Starch Improved the Gut Barrier Function of Depressed Mice

**Figure 3:**
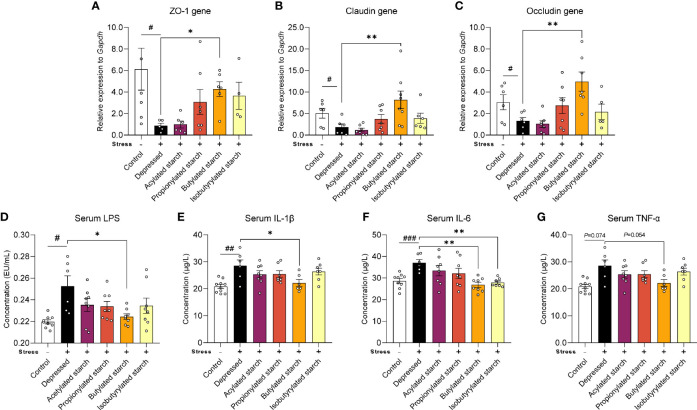
The SCFAs levels in the ileum and cecum. **(A–C)** mRNA levels of the ZO-1, Claudin, and Occludin protein in the colon tissues. **(D–G)** Serum LPS and inflammatory cytokines (IL-1β, IL-6, and TNF-α) levels. Except for the control, all groups are given two-week chronic restraint stress. Data are mean with SEM (n=6-10 per group for each test). ^#^ *P*<0.05, ^##^ *P*<0.01, ^###^ *P*<0.001 in the unpaired t-tests. *P<0.05, **P<0.01 in the unpaired t-tests.

Gut barrier function was evaluated by the gene expression of tight junction protein levels. CRS induced a significant reduction of the transcriptional level of the ZO-1 gene *P*=0.049), the Claudin gene (*P*=0.038), and the Occludin gene (*P*=0.048). Intriguingly, the butylated starch showed a superior effect to others to restore the gut barrier (**Figures 3A–C**). The deficit of gut barrier resulted in increased serum lipopolysaccharide (LPS; **Figure 3D**) and inflammatory cytokines, including serum IL-1β (*P*=0.001), IL-6 (*P*<0.001), and TNF-α (*P*=0.074) (**Figures 3E–G**). Butylated starch significantly reduced the serum IL-1β (*P*=0.025) and IL-6 (*P*=0.002) levels. The isobutyrate starch could also reduce the serum IL-6 level (*P*=0.036; **Figure 3F**). In addition, butylated starch reduced the serum TNF-α level, with a trend statistical difference (*P*=0.054, **Figure 3D**).

### Butylated Starch Changed the Gut Microbial Composition and Function of Depressed Mice

**Figure 4:**
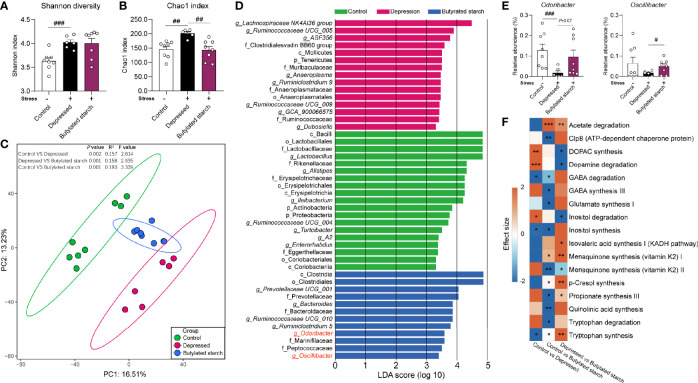
The SCFAs levels in the ileum and cecum. **(A, B)** Alpha diversity of the gut microbiome, quantified using the Shannon and Chao 1 index. Except for the control, all groups are given two-week chronic restraint stress. Data are mean with SEM (n=6-10 per group). ^##^ *P*<0.01, ^###^ *P*<0.001 in the unpaired t-tests. **(C)** Beta diversity of the gut microbiome. PCA based on the center-log ratio transferred Aitchison distance followed by the PERMANOVA. **(D)** Microbial biomarkers are identified by the linear discriminant analysis (LDA) effect size algorithm (LEfSe). α<0.05 in the Wilcoxon rank-sum test and log LDA>2.0 were used as the threshold. **(E)** Relative abundance of selected microbes. Data are mean with SEM (n=6-10 per group). ^#^ *P*<0.05, ^###^ *P*<0.001 in the unpaired t-tests. **(F)** Gut-brain module analysis. The colors of the boxes indicate the effect size between the two groups. Welch’s t-test was performed between two groups. Asterisks in the heat map represent the Benjamini-Hochberg false discovery rate (FDR)-adjusted p-value: *P<0.05, **P<0.01, ***P<0.001.

CRS induced a significantly increased gut microbial alpha-diversity, as reflected by the Shannon (*P*<0.001) and Chao1 (*P*=0.001) index (**Figures 4A, B**). The butylated starch treatment normalized the Chao1 changes (*P*=0.003; **Figure 4B**). Besides, the beta diversity estimated using Aitchison distance and principal component analysis (PCA) showed that every two groups are significantly different (PERMANOVA results in **Figure 4C**). The linear discriminant analysis (LDA) effect size algorithm (LEfSe) identified five genus-level biomarkers of the butylated starch treated gut microbiome, including *Bacteroide*, *Ruminiclostridium*, *Odoribacter*, and *Oscillibacter* (**Figure 4D**). The relative abundance of these four taxa was all significance decreased under stress, and the butylated starch treatment increased the *Odoribacter* (*P*=0.072) and *Oscillibacter* (*P*=0.048) abundances (**Figure 4E**). The microbial genomic functions were analyzed based on the gut-brain modules as previously described (22). A total of 17 pathways were identified, and four of them are significantly affected by the CRS and reversed by the butylated starch treatment, including *striatal dopamine (DA) and metabolite (DOPAC) synthesis*, *Dopamine degradation*, *Inositol degradation*, and *Tryptophan synthesis* (**Figure 4F**).

## Discussion

In the present study, oral intake of SCFA-acylated starches could significantly increase the SCFAs levels in the large intestine, which improves the neurobiological conditions of the mice enduring chronic stress. In particular, the butylated starch could significantly enhance the gut barrier integrity and reduce inflammation. The butylated starch also improved the gut microbial composition and function, contributing to the recovery from the stress-induced disorder.

Although accumulated research have proved the beneficial role of SCFAs in regulating brain function, using dietary SCFAs to address psychological disorders did not make good progress (7). Since most SCFAs have a strong smell, the direct intake of SCFAs could cause olfactory discomfort and may affect neurobehavioral performance (23). Moreover, oral intake SCFAs are primarily absorbed by the upper intestine, which cannot mimic the natural production of SCFAs in the large intestine (24, 25). Since current evidence that supports the SCFAs’ health effect is mainly generated from the research focused on the large intestine, direct oral intake of the SCFAs may not be the optimal choice (26). Intaking dietary fibers is another widely used way to enhance the SCFAs’ production in the large intestine (27, 28). However, the production of each SCFA cannot be quantitatively controlled, and the role of each SCFA is hard to identify.

Accumulating studies demonstrated that acylated starch has small enzyme resistance and could reach the large intestines (29, 30). Here, a catalytic reaction of corn starch with SCFA anhydrides was used to obtain the SCFA-acylated starches (31). These starches cannot be digested and absorbed when the degree of substitution was modified to the range of 0.2 to 0.3 (32). When reaching the large intestine, they are fermented by the gut microbiome and release the free SCFAs (16, 32). Our results reproduced the phenomenon, reflected as the higher SCFA levels in the cecum than in the ileum (**Figure 2**). Also, each starch only significantly increased the corresponding acid without affecting other acids’ levels (**Figure 2**). Although not very precise, to some extent, the administration of SCFA-acylated starches realize a sizeable intestine-targeted release and elevation of each SCFA, which offers a great model for us to further investigate the SCFA’s psychotropic effects.

A neurobehavioral change of stressed mice was observed in the acetylated-, butylated-, and isobutylated- starch treated mice, but only butylated starch significantly improved the gut barrier function and immune status. To investigate the underlying mechanisms, the gut microbiome was analyzed, and some interesting findings were observed. Butylated-starch treatment established a novel microbial structure that differs from healthy and depressed mice (**Figure 4C**). Specifically, the reduced *Odoribacter* and *Oscillibacter* abundance was largely recovered (**Figure S2**). *Odoribacter* is a common SCFA-producing microbe, and the deficit of *Odoribacter* has been correlated to many metabolic and immunological disorders, including chronic kidney disease (33), Crohn’s disease (34), and inflammatory bowel disease (35). A recent study proved that *Odoribacter splanchnicus* has very low adherent and inflammatory capacity to the enterocytes or mucus and can secret the outer membrane vesicles that exert anti-inflammatory action in the gut epithelium (36).

*Oscillibacter* is another microbial taxa that sensitively responded to stress (37, 38), and the abundance is found to be significantly decreased in significant depression disorder patients (39). In addition, the butylated-starch modified microbial function also facilitates the neurotransmission function through upregulating the *Tryptophan synthesis* and downregulating the *Inositol degradation* pathways. Tryptophan is the precursor of serotonin, which is a crucial neurotransmitter that regulating multiple brain functions. Inositol was widely verified to be efficacious in treating depression and obsessive-compulsive disorder, and the mechanism correlates to the enchantment of serotonin production *via* the 5-HT2 receptors (40, 41). Collectively, normalizing the key microbes and metabolomic pathways seems beneficial to the host’s immune and brain function.

## Conclusions

In conclusion, the butylated starch could alleviate the chronic restraint stress-induced neurobehavioral and gut barrier deficits. The mechanisms may link to the modification of gut microbial composition and function. Butylated starch’s anti-depressive and anti-inflammatory effects further validate the beneficial role of SCFAs in host health and offer novel guidance for developing novel food or dietary supplements for improving mental health.

## Data Availability Statement

The original contributions presented in the study are publicly available. This data can be found here: https://www.ncbi.nlm.nih.gov/geo/query/acc.cgi?acc=GSE182262.

## Ethics Statement

The animal study was reviewed and approved by Ethics Committee of Experimental Animals at Jiangnan University (JN.No20190930c0501205[256]).

## Author Contributions

PT: conceptualization, investigation, writing-original draft, and funding acquisition. HuZ: investigation and formal analysis. YC, XQ, and ZW: formal analysis and visualization. HaZ and JZ: methodology, resources, and writing-review and editing. WC and GW: supervision and funding acquisition. All authors contributed to the article and approved the submitted version.

## Funding

This work was financially supported by the National Natural Science Foundation of China (No. 31972052, 32021005, 31820103010), the Fundamental Research Funds for the Central Universities (JUSRP22006, JUSRP51501), the China National Postdoctoral Program for Innovative Talents (BX2021114), China Postdoctoral Science Foundation (2021M691290), the Postdoctoral Science Foundation of Jiangsu Province (2021K127B), the Program of Collaborative Innovation Centre of Food Safety and Quality Control in Jiangsu Province.

## Conflict of Interest

The authors declare that the research was conducted in the absence of any commercial or financial relationships that could be construed as a potential conflict of interest.

## Publisher’s Note

All claims expressed in this article are solely those of the authors and do not necessarily represent those of their affiliated organizations, or those of the publisher, the editors and the reviewers. Any product that may be evaluated in this article, or claim that may be made by its manufacturer, is not guaranteed or endorsed by the publisher.
