# Supplementary material for: Consumption of Butylated Starch Alleviates the Chronic Restraint Stress-Induced Neurobehavioral and Gut Barrier Deficits Through Reshaping the Gut Microbiota
Source: Front Immunol. 2021 Sep 17;12:755481. doi: 10.3389/fimmu.2021.755481 (PMC8485752; doi:10.3389/fimmu.2021.755481)
Supplement: Supplementary file 1 [file DataSheet_1.docx]

Supplementary Material

# Supplementary Methods

## Degree of substitution of the SCFA-acylated starch

The degree of substitution (DS) was determined by acid-base titration, during which releasing SCFAs. DS was calculated using the following formula:

$$A=\left( \frac{V2}{m2}-\frac{V1}{m1} \right)\times\frac{c\times M\times100}{1000}$$

$$DS=\frac{162\times A}{100\times M-（M-1）\times A}$$

Where A is acyl content of the sample, %; V1 is volume of HCl standard solution consumed by acylated starch, mL; V2 is volume of HCl standard solution consumed by normal starch, mL; c is concentration of hydrochloric acid standard solution, mol/L; m1 is mass of acylated starch, g; m2 is mass of normal starch, g; M is the molar mass of the substituent (g/mol, 43 for acetyl, 57 for propanoyl, 71 for butyryl and isobutyryl); 162 is the molecular weight anhydroglucose unit.

The DS of the acetylated starch (AS), propionylated starch (PS), butyrylated starch (BS), isobutyrylated starch (IBS) was 0.22, 0.25, 0.26 and 0.22. Using high amylose corn starch (Ingredion Incorporated, USA) as normal starch control, the control group and the depression group were feed a diet with 15% normal starch. The AS, PS BS, IBS groups were respectively feed with 15% acylated starch for 2 weeks during chronic restraint stress.

## Bioinformatical analysis

Bioinformatical analysis was performed as previously described (*Molecular Nutrition & Food Research*, 2021, 65(8): 2000704). The raw data of sequencing was processed using Quantitative Insights into Microbial Ecology (QIIME) wrapper and software (Caporaso, J, et al. Nature methods 2010, 7, 335).The operational taxonomic unit (OTU) was picked using a criterion of 97% nucleotide identity and the taxonomy was assigned using the SILVA database. The Shannon and Chao 1 indices were measured from the rarefied OTU. β-diversity was estimated using compositional data analysis by PCA using Aitchison distance (Gloor, G. B, et al. Front. Microbiol. 2017, 8, 2224), following a PERMANOVA test to assess differences. LEfSe was used to differentiate microbial taxa (Wilcoxon rank-sum test, α < 0.05 and log LDA>2.0 were used as the threshold). GBMs analysis was performed as previously described (Valles-Colomer, M, J, et al. Nature microbiology 2019, 4, 623). Briefly, the metagenomic composition was predicted from 16S rRNA sequences using PICRUST2 (Nature Biotechnology, 2020, 38(6): 685-688). The identified KEGG Orthogroups (KOs) abundance was normalized and mapped to 56 GBMs using the Omixer-RPM V1.0. The GBMs enrichment differences were calculated by Welch's t-test, *P*<0.05 adjusted by the Benjamini & Hochberg method were considered significantly different.

# Supplementary Figures and Tables

## Primers information of the qRT-PCR

**Table S1 Primers information**

| Primer name | Sequence (5' - 3') |
| --- | --- |
| *Gapdh* | F-5’-AGGTCGGTGTGAACGGATTTG-3’ |
|  | R-5’TGTAGACCATGTAGTTGAGGTCA-3’ |
| *Claudin-1* | F-5’-CACTTCCAGACTCCACCACC-3’ |
|  | R-5’-AATCTTCCATTGGGGCAGGG-3’ |
| *Occludin* | F-5’-CACACTTGCTTGGGACAGAG-3’ |
|  | R-5’-TAGCCATAGCCTCCATAGCC-3’ |
| *ZO-1* | F-5’-CTTCTCTTGCTGGCCCTAAAC-3’ |
|  | R-5’-TGGCTTCACTTGAGGTTTCTG-3’ |

## Supplementary Figures


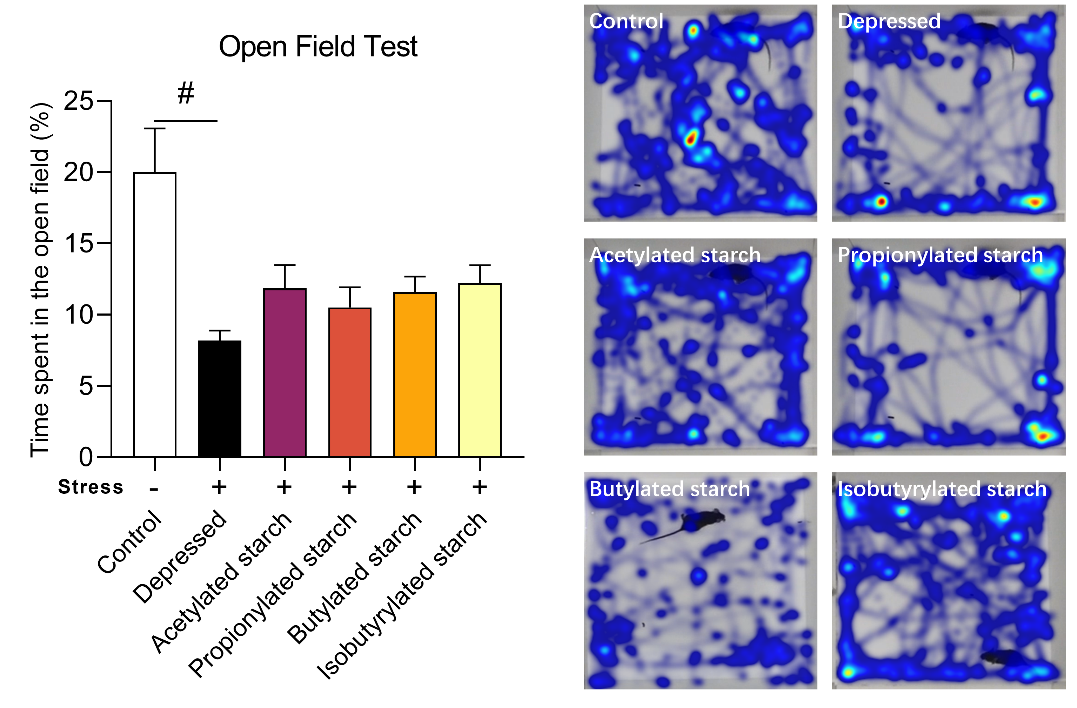


**Figure S1.** Open field test. Except for the control, all groups are given two-week chronic restraint stress. Data are mean with SEM. ^#^*P*<0.05 in the unpaired t-tests.
